# Analysis of a deep learning-based method for generation of SPECT projections based on a large Monte Carlo simulated dataset

**DOI:** 10.1186/s40658-022-00476-w

**Published:** 2022-07-19

**Authors:** Julian Leube, Johan Gustafsson, Michael Lassmann, Maikol Salas-Ramirez, Johannes Tran-Gia

**Affiliations:** 1 Department of Nuclear Medicine, University of Würzburg, Oberdürrbacher Str. 6, 97080 Würzburg, Germany; 2 Medical Radiation Physics, Lund, Lund University, Skåne University Hospital, Lund, 221 85 Lund, Sweden

**Keywords:** ^177^Lu, Monte Carlo, SPECT, Deep learning, Denoising

## Abstract

**Background:**

In recent years, a lot of effort has been put in the enhancement of medical imaging using artificial intelligence. However, limited patient data in combination with the unavailability of a ground truth often pose a challenge to a systematic validation of such methodologies. The goal of this work was to investigate a recently proposed method for an artificial intelligence-based generation of synthetic SPECT projections, for acceleration of the image acquisition process based on a large dataset of realistic SPECT simulations.

**Methods:**

A database of 10,000 SPECT projection datasets of heterogeneous activity distributions of randomly placed random shapes was simulated for a clinical SPECT/CT system using the SIMIND Monte Carlo program. Synthetic projections at fixed angular increments from a set of input projections at evenly distributed angles were generated by different u-shaped convolutional neural networks (u-nets). These u-nets differed in noise realization used for the training data, number of input projections, projection angle increment, and number of training/validation datasets. Synthetic projections were generated for 500 test projection datasets for each u-net, and a quantitative analysis was performed using statistical hypothesis tests based on structural similarity index measure and normalized root-mean-squared error. Additional simulations with varying detector orbits were performed on a subset of the dataset to study the effect of the detector orbit on the performance of the methodology. For verification of the results, the u-nets were applied to Jaszczak and NEMA physical phantom data obtained on a clinical SPECT/CT system.

**Results:**

No statistically significant differences were observed between u-nets trained with different noise realizations. In contrast, a statistically significant deterioration was found for training with a small subset (400 datasets) of the 10,000 simulated projection datasets in comparison with using a large subset (9500 datasets) for training. A good agreement between synthetic (i.e., u-net generated) and simulated projections before adding noise demonstrates a denoising effect. Finally, the physical phantom measurements show that our findings also apply for projections measured on a clinical SPECT/CT system.

**Conclusion:**

Our study shows the large potential of u-nets for accelerating SPECT/CT imaging. In addition, our analysis numerically reveals a denoising effect when generating synthetic projections with a u-net. Clinically interesting, the methodology has proven robust against camera orbit deviations in a clinically realistic range. Lastly, we found that a small number of training samples (e.g., ~ 400 datasets) may not be sufficient for reliable generalization of the u-net.

**Supplementary Information:**

The online version contains supplementary material available at 10.1186/s40658-022-00476-w.

## Introduction

Quantitative SPECT is the basis for patient-specific dosimetry in radionuclide therapy (RNT), which, in turn, can be used for individualization of the treatment and for improved understanding of biological effects [1]. In order to increase patient comfort and reduce the risk of motion artifacts, there is a striving to shorten acquisition protocols while at the same time maintain a sufficient signal-to-noise ratio (SNR). This is particularly important when using multiple bed positions in order to cover a large axial field of view. Due to the recently reported therapeutic successes of ^177^Lu-based radiopharmaceuticals in the treatment of neuroendocrine tumors (^177^Lu-DOTATATE, [2]) and castration-resistant prostate cancer (^177^Lu-PSMA, [3, 4]), quantitative SPECT of ^177^Lu plays an increasingly important role for planning and monitoring of RNTs.

With the advent of artificial intelligence (AI) and—more specifically—neural networks in the field of medical imaging, there have recently been attempts to acquire less signal (e.g., by reducing the acquisition time) and compensate for the resulting signal loss (i.e., the decreasing SNR) by applying convolutional neural networks trained either based on simulated or based on clinical SPECT data.

In a recent review article on the applications of AI in SPECT imaging [5], Arabi et al. divide AI-based solutions in this field into two groups: (i) techniques replacing current algorithms or frameworks due to their superior performance and (ii) approaches that enable tasks that are not solvable using conventional methods. Approaches of the first category directly compete with the existing methods, making them easy to assess. While the second category, into which the methodology investigated in this paper falls, does present new opportunities for improved SPECT imaging, such methods also require extensive validation using large clinical databases and a wide range of conditions. This far, AI-based approaches for acceleration of SPECT imaging have demonstrated an enormous potential. However, more validation is required in order to justify a widespread clinical adoption of any of the presented techniques.

This study aims to generate a large as realistic as possible training dataset based on Monte Carlo (MC) simulations for a clinical SPECT setup. To systematically test the functionality of the proposed u-shaped convolutional neural network (u-net) presented by Rydén et al. [6], a database consisting of 10,000 MC simulated projection datasets of heterogeneous activity distributions was generated using the SIMIND MC program [7]. Using this database of projection datasets, various u-nets were trained to generate projections missing from a subset of the original projection datasets of 120 projections (e.g., every other projection was initially omitted and these projections were then generated using the u-net). To test how different factors influence the performance of the neural network, the amount of training data, number of input projections, and the noise realization were varied to create different versions of the trained u-net. Finally, our simulation-based observations were validated for ^177^Lu SPECT/CT data of a Jaszczak cylinder and an IEC NEMA body phantom (“NEMA phantom”) with a six-sphere insert, which had been acquired on a clinical SPECT/CT system.

## Methods

**Fig. 1:**
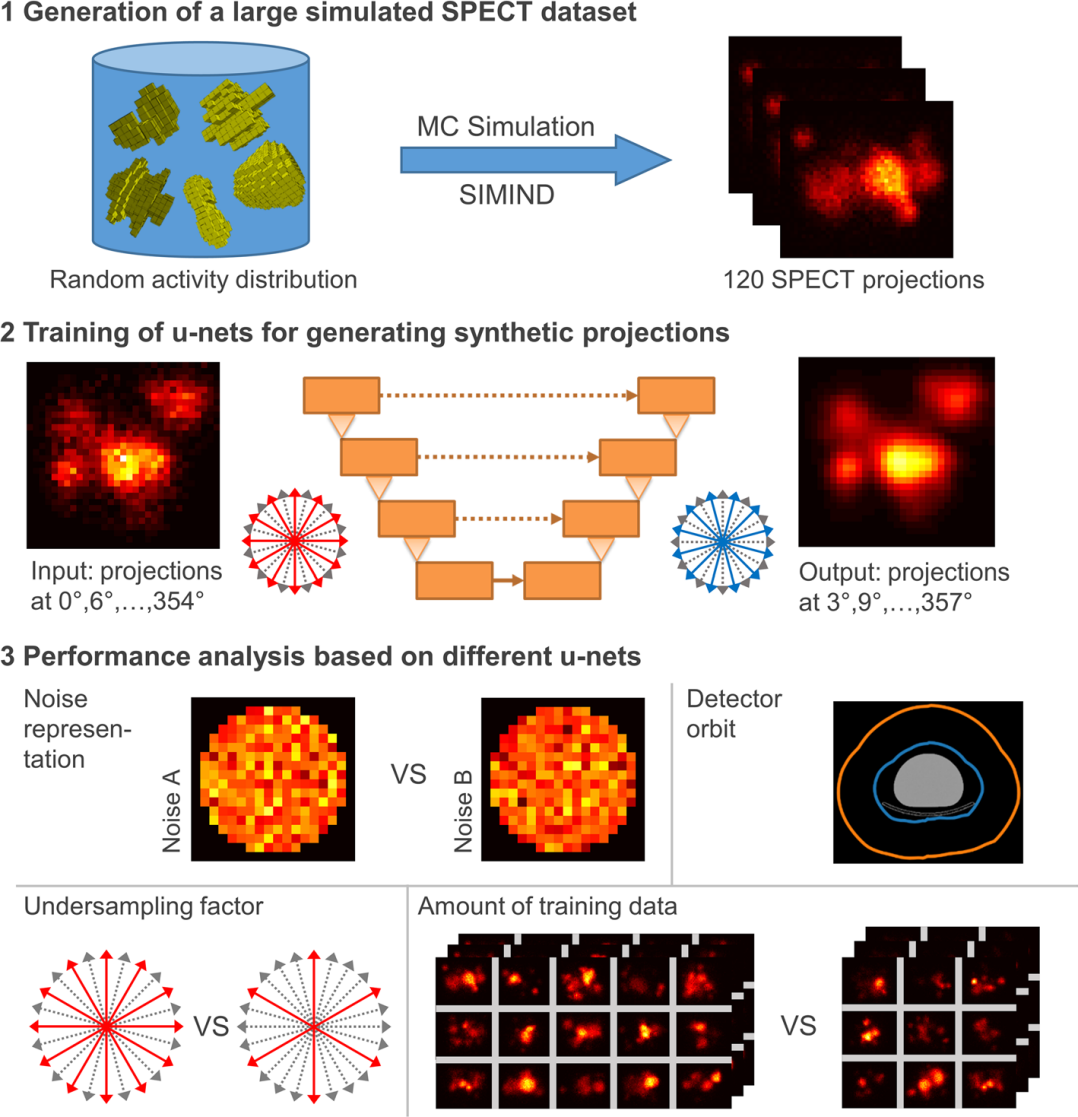
Schematic representation of the experimental setup and analysis

Figure 1 shows an overview of the experimental setup used in this work. The individual steps will be explained in the following sections.

### Generation of a training dataset of simulated SPECT projections

**Fig. 2:**
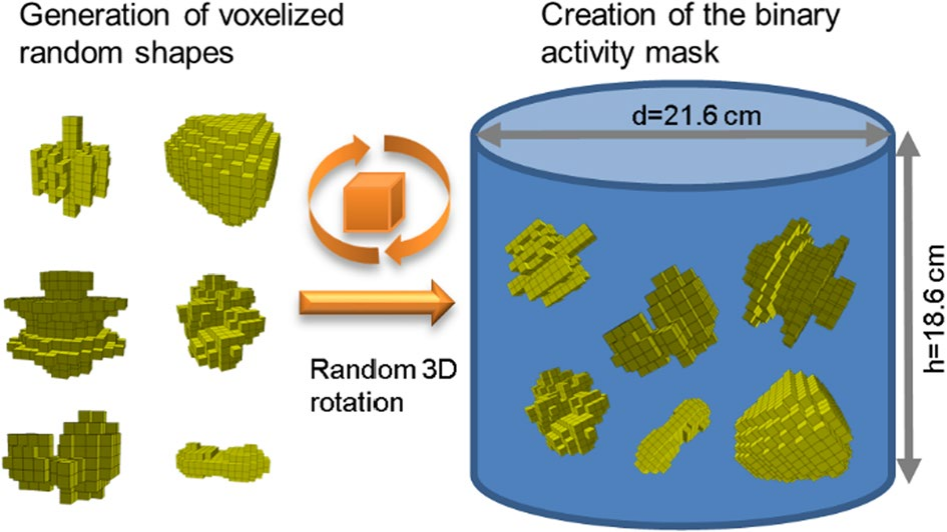
Illustration of the creation of the binary activity masks. For each binary activity mask, a set of random shapes of different sizes is generated. Afterwards, a random 3D rotation is performed for each shape and it is placed at a random location inside the Jaszczak phantom

**Fig. 3:**
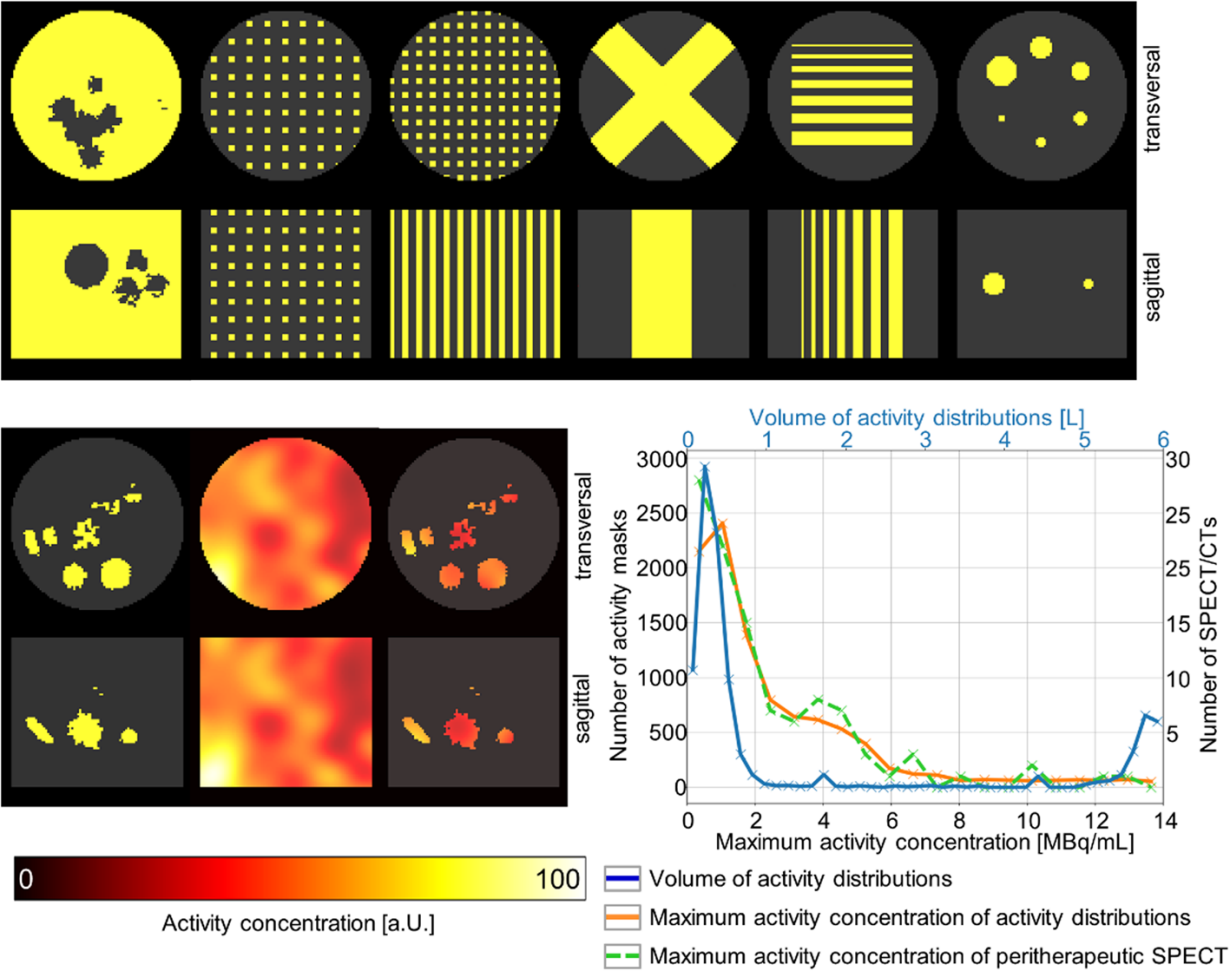
Illustration of the activity mask dataset. For each activity mask, the transversal (top) and sagittal sections (bottom) are shown. First row from left to right: hot Jaszczak with cold random shapes, chessboard pattern, rod pattern, cross pattern, stripe pattern, NEMA phantom spheres. Second row from left to right: example random shape, example Fourier series *F*(*x*, *y*, *z*), random shape with non-uniform activity distribution. The plot shows the distribution of the maximum activity concentration (orange, bottom and left axes) and volumes (blue, top and left axes) of the activity distributions contained in the dataset. The dashed green curve represents the distribution of the maximum activity concentration of 104 peritherapeutic ^177^Lu SPECT/CT examinations (bottom and right axes)

Three-dimensional (3D) activity distributions of randomly arranged random shapes were generated as a basis to create a large database of projection datasets for training and assessment of different neural networks (Fig. 2). These voxelized 3D shapes were constructed with a random shape generator based on (i) a sphere perturbed with spherical harmonics [8], (ii) a 3D implementation of the super-formula [9], or (iii) volumetric Perlin noise with randomized parameters [10]. For each simulation, 10 to 25 of these shapes with volumes ranging from 8 to 64 voxels (voxel size 2.4 × 2.4 × 2.4 mm^3^) were chosen, randomly rotated in 3D, and then placed in a cylindrical water phantom (Jaszczak without inserts, diameter 21.6 cm, length 18.6 cm, volume 6.8 L). The result was a 256 × 256 × 256 binary activity mask that described the presence of activity in each voxel (0: no activity, 1: activity). An illustration of the generation of the binary activity masks is shown in Fig. 1. While the majority of the mask dataset (7500) consisted of these random activity distributions, a few special cases (2500) were added to further expand the diversity of the dataset, as further specified in Table 1. An illustration of the activity mask dataset is shown in Fig. 3. In addition, the graph in the lower right of Fig. 3 shows the distribution of the volumes of all 10,000 activity masks (solid blue curve). To generate realistic projections of the activity masks, MC-based SPECT simulations were performed using the SIMIND MC program [7]. The simulated system was a Siemens Intevo Bold SPECT/CT system with a 9.5-mm crystal, medium-energy collimator (Siemens medium-energy low penetration, MELP), and 9% energy resolution.

**Table 1:** Composition of the activity mask dataset (total of 10,000 masks)

Name of mask	Description	Sample size
Random shapes	Voxelized random shapes	7500
Hot Jaszczak with cold random shapes	Inverted version of *Random shapes* masks. Here, the entire Jaszczak cylinder with the exception of the random shapes is filled with activity	2000
Chessboard pattern	Randomly sized squares placed on a randomly sized isotropic 3D grid	100
Rod pattern	Rods with randomly large cross sections placed perpendicular to the transverse plane on a randomly sized isotropic 2D grid	100
Cross pattern	Randomly sized cross extended along the axial direction	100
Stripe pattern	Seven stripes with a thickness varying between 2.4 and 16.8 mm placed perpendicular to the transverse plane	100
NEMA phantom spheres	Voxelized activity distribution of the NEMA phantom (six fillable spheres with inner diameters 10/13/17/22/28/37 mm)	100

Before inclusion in the mask, all objects were randomly rotated in the three spatial dimensions (exception: NEMA phantom, random two-dimensional rotation in the transversal plane)

To increase the realism of the simulated SPECT projections, the binary activity masks were transformed into heterogeneous activity distributions by a voxel-by-voxel multiplication with a spatially contiguous, non-uniform pattern. This function *F*(*x*, *y*, *z*) was constructed as Fourier series according to

1 $$F\left( {x,y,z} \right) = \sum\limits_{k = - M}^{M} {\sum\limits_{j = - M}^{M} {\sum\limits_{l = - M}^{M} {g_{k,j,l} \frac{{ \cos \left( { \frac{{2\pi \left( {kx + jy + lz} \right)}}{p} + \varphi_{k,l,j} } \right)}}{{\left( {k^{2} + j^{2} + l^{2} } \right)^{\beta } }}} } } ,$$

where $g_{k,j,l}$ is a random Gaussian distributed number (mean = 0, SD = 1), $\varphi_{k,l,j}$ is a random phase between $- \pi { }$ and ${ }\pi$, and (*x*, *y*, *z*) is the position of the voxel [11]. Here, the parameter β determines how quickly higher frequencies are attenuated, $p$ is the period of $F$, and $M$ is the spatial cutoff frequency. To create a realistic activity distribution within a reasonable calculation time, the following parameters were empirically chosen: *β* was set to 0.9 according to [11], M was set to 8 to achieve a complex surface in a reasonable calculation time, and the period p was set to 50 voxels, so that the dimension of the activity distribution covered the dimension of the Jaszczak cylinder. After applying this function to the binary activity masks, the results were rescaled to integers between 0 and 100 (100: highest activity concentration, 0: no activity).

The resulting activity distributions served as input for the SPECT simulations with an analytical water-filled cylinder (Jaszczak dimensions) as attenuating and scattering medium. The simulations mimicked a SPECT acquisition with 120 projections of 22 s each, matrix size 128 × 128, pixel size 4.79 × 4.79 mm^2^, and a 20% main energy window at 208 keV following a non-circular orbit based on the one from a physical phantom measurement of the NEMA phantom. An analytical expression was used for modeling the MELP collimator using the specifications provided by the manufacturer [12], thereby excluding penetration and scattering in the collimator from the simulation. The simulations of all 10,000 projection sets were performed on a local high-performance computing cluster (High Performance Computing Cluster, University of Würzburg). The SIMIND MC program uses a number of variance reduction techniques to speed up simulations [13]. As a consequence, the MC noise in the simulated projections is not representative of the noise of the corresponding physical measurement. Hence, the normal mode of operation is to use a large number of histories in the simulations in order to achieve essentially noise-free projections (i.e., residual MC noise negligible compared with noise in a real measurement), which are then scaled to the desired projection time and activity before adding Poisson-distributed noise. The simulated SPECT projections *before* and *after* the addition of Poisson noise will be referred to as *noise-free* and *noisy* projections, respectively. The output of the simulations was scaled to maximum activity concentrations between 0.2 MBq/mL and 14 MBq/mL. This distribution was based on the maximum activity concentration of 104 peritherapeutic SPECT/CT examinations, which had been performed as part of ^177^Lu-PSMA-based endoradiotherapy at University Hospital Würzburg (DICOM Tag (0028,0107): Largest Image Pixel Value). The distributions of maximum activity concentrations of patients (dashed green curve) and heterogeneous activity distributions (solid orange curve) are illustrated in the graph in Fig. 3.

### U-net architecture

A u-shaped 3D convolutional neural network (u-net) design was used to generate “synthetic” SPECT projections (output) based on “original” SPECT projections (input). Technically, the u-net was trained to calculate intermediate projections at shifted projection angles (e.g., shifts of 3°, 6°, or 9° for a circular arch of 12° between two projections in the input dataset, used to generate 3 × 30 projections from an input dataset of 30 projections) with respect to the original projections. For better readability, the technically imprecise terms “rotation of the projections” and “rotated projections” will be used when referring to these intermediate projections. As an example, u-net U1 was trained to rotate 60 original projections by 3° to generate 60 synthetic projections (0°, 6°, …, 354° → 3°, 9°, …, 357°).

The u-nets were based on the fastMRI architecture [14] and were implemented using the PyTorch library [15]. The network consists of two main components: a down-sampling path that compresses image information and acts as an encoder, and an up-sampling path that reconstructs the image from the compressed data and thus acts as a decoder. Both paths consist of four convolutional blocks, each executing two 3 × 3 × 3 3D convolutions with instance normalization and leaky rectified linear unit (leaky ReLU) activation function. In the encoder path, the number of channels is doubled after each convolutional block, whereas in the decoder path, the number of channels is halved. Skip connections between opposing blocks of the two paths ensure that the decoder can reconstruct an image using fine-grained features learned in the encoder phase. After each convolutional block, the image size in each spatial dimension is halved using a max-pool operation with stride 2 in the encoder phase. In contrast, the image size in each spatial dimension is doubled after each convolutional block in the decoder phase using transposed convolutions with kernel size 2 × 2 × 2 and stride 2. At the end of the up-sampling path, two 1 × 1 × 1 convolutions with ReLU activation function are performed to reduce the number of channels to one while maintaining the size of the image. An illustration of the u-net’s architecture can be found in Additional file 1.

### Generation of a set of u-nets trained with different parameters

To understand the underlying mechanisms of the generation of synthetic projections by the u-net (with and without rotation), seven different u-nets were trained (U1–U7). Their properties such as the number of input and output projections, the size of the training and validation datasets, the Poisson noise realization used, and by which angle $\theta$ the output projections were rotated relative to the input projections are listed in Table 2. As an example, u-net U1 was trained after adding Poisson noise realization A to 9,500 noise-free datasets. As described above, training was performed based on 60 input and output projections with a rotation of 3° between each input and output. To force a cyclical projection dataset, the first and last projections were added to the back and the front of the projection series, respectively, until the integer power of 2 was reached (e.g., the first 2 and the last 2 of 60 available projections [1, 2, …, 59, 60] were added at the front and at the back, respectively, to reach a total of 64 projections [59, 60, 1, 2, …, 59, 60, 1, 2]). This was done to ensure that there is an original projection on both sides of all intermediate projections to be generated, thereby avoiding extrapolation. Prior to being used as input for the u-net, each projection dataset was normalized to an interval between 0 and 1 by dividing each voxel by the maximum voxel value of the respective input projections. After applying the u-net, the projections were rescaled with the same value. The dataset was then separated into training and validation datasets of sizes 9000 and 500, respectively. Training was performed for 60 epochs using an Adam optimizer [16], a mini-batch size of 5 and an L1 loss function. The initial learning rate was set to 7 × 10^−5^, which was halved every 20 epochs. After every epoch, the mean L1 loss on the validation dataset was calculated and the network weights with the lowest validation loss were saved.

**Table 2:** Overview over all u-nets trained in this study

Abbreviation	Poisson noise realization	Number of input/output projections	Cyclical expansion	Rotation shift	Size of training/validation dataset
U1	A	60	64	3°	9000/500
U2	B	60	64	3°	9000/500
U3	A	60	64	3°	400/40
U4	A	30	32	3°	9000/500
U5	A	30	32	6°	9000/500
U6	A	30	32	9°	9000/500
U7	A	120	120	0°	9000/500

### Influence of noise

To investigate the influence of noise on the u-net performance, two different u-nets U1 and U2 with different Poisson noise realizations A and B, created by using different seeds for the random number generator, were trained (Table 2).

### Influence of the size of the training dataset

To examine the effect of the amount of training data on the performance of the u-net, a third u-net, U3, was trained (Table 2), where the sizes of the training/validation datasets were similar to the training/validation dataset size of 352/37 used by Rydén et al. [6]. This was achieved by selecting 400/40 projection datasets from the training/validation datasets of u-net U1. The proportions of the different types of activity masks (Table 1) were retained. To ensure convergence of the training, the number of epochs was increased to 200 and a linearly decreasing learning rate from 1.2 × 10^−5^ to 0.8 × 10^−5^, as described in [6], was selected.

### Influence of the number of input projections and rotation angle

To assess whether the number of input projections has an impact on the performance of the methodology, three additional u-nets were trained (U4, U5, and U6). Each of these u-nets generates 30 intermediate projections at differently shifted projection angles (3°, 6°, and − 3°) with respect to the 30 input projections.

### Analysis of the u-net for denoising the projections

To determine whether and to what extent the synthetically created projections of u-nets U1 to U6 are denoised, an additional u-net U7 was trained. This network was trained to create 120 noise-free output projections from 120 noisy input projections, i.e., without shifting the projection angles between input and output (“no rotation”).

### Evaluation criteria for quantifying the u-net performance

For all trained u-nets, a quantitative analysis was performed based on the same test dataset consisting of 500 projection sets with noise realization A. To determine the agreement between synthetic (e.g., projections for 3°, 9°, …, 357° for u-net U1) and ground-truth projections (i.e., acquired for the same angles as the output projections), the structural similarity index measure (SSIM) [17] and normalized root-mean-squared error (NRMSE) were calculated for all test data. The synthetic projections (i.e., generated by the u-net) were compared to both the noise-free and the noisy projections to assess and quantify the denoising effect of the methodology. Each comparison was made based on statistical hypothesis tests between the SSIM or NRMSE values of two u-nets to be compared. Since the NRMSE values are normally distributed (Shapiro–Wilk test), a paired two-sided t test was performed. For the non-normally distributed SSIM values, a paired two-sided Wilcoxon signed-rank test was performed. The following sections describe the aspects investigated with the various u-nets (see Table 2).

### Influence of the detector orbit

**Fig. 4:**
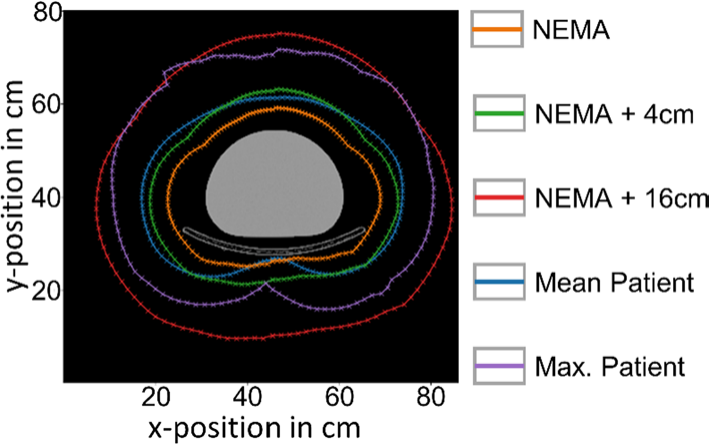
CT of NEMA body phantom placed on patient bed with different gamma camera orbits. The orange contour illustrates the original NEMA detector orbit, which is used for the simulations of all activity distributions in the dataset. The green and the red contours represent two expanded versions of the NEMA detector orbit, used for analyzing the influence of deviations in detector orbit between training and input data on the u-net performance. The blue and purple contours represent the mean and the maximum (maximum distance for every angle) patient detector orbits for all 436 SPECT/CT scans

All projection data used for training the u-nets were simulated with identical detector orbits. Therefore, it could be suspected that the generation of synthetic projections works only for SPECT images with that specific detector orbit. To determine the impact of the detector orbit on the performance of the u-nets, four additional MC simulations were run for each activity distribution in the test dataset. For these simulations, the distance of the detector at all angular positions was increased by 2 cm, 4 cm, 8 cm, and 16 cm, respectively, compared to the original NEMA detector orbit. To get an impression of the extent of detector orbit variations in real patient examinations, the orbits of a total of 436 ^177^Lu-PSMA-based SPECT/CT examinations performed at University Hospital Würzburg were analyzed. For each angle, both the mean and the maximum distance of the gamma camera to the center were determined. The results are shown in Fig. 4 together with the NEMA detector orbit and its expanded versions (4 cm and 16 cm). The mean and maximum patient detector orbits have similar projection distances as the NEMA orbit enlarged by 4 cm and 16 cm, respectively.

Based on each of these four new detector orbits, synthetic projections were generated using u-net U1, which had been trained with data from the original orbit (i.e., without radial expansion). These synthetic projections were compared with the corresponding ground-truth projections (i.e., noisy simulations for the respective detector orbit).

### Physical phantom-based verification of the simulation-based findings

Two phantom measurements were performed with the same clinical SPECT/CT system that had been used as template for the MC simulations. In the first measurement, a large Jaszczak cylinder without inserts was filled with a uniform ^177^Lu stock solution (activity concentration 59.9 kBq/mL). The second measurement was a water-filled NEMA phantom equipped with six ^177^Lu-filled spheres (activity concentration 1.99 MBq/mL). Both experiments were performed using a MELP collimator, 180° head configuration, auto-contouring, continuous mode, 60 views, 30 s per view, 128 × 128 matrix, and a 20% energy window around the 208 keV photopeak. After each SPECT acquisition, two low-dose CT scans were acquired (tube voltage 130 kV, 26–30 mAs, 1.0 × 1.0 mm^2^ in-plane pixel size, 1.5 pitch): in addition to a standard low-dose CT acquisition for attenuation correction (3.0 mm slice thickness), a high-resolution low-dose CT (1.0 mm slice thickness) was acquired for determining the phantom positioning.

The measurements were replicated in SIMIND as follows: The centers of the spheres of the NEMA phantom and the filled Jaszczak cylinder were determined using the high-resolution CT. Two simulations were then performed in SIMIND using the known dimensions of both phantoms (diameter of the NEMA spheres: 10, 13, 17, 22, 28, 37 mm; height and diameter of the Jaszczak cylinder: 186 and 216 mm). Attenuation and scatter were simulated based on the CT images of the physical phantom measurements: First, the attenuation CT was scaled by linear interpolation to the standard resolution of the simulations performed (256 × 256 matrix, 2.4 × 2.4 × 2.4 mm^3^ voxel size). Hounsfield units were then converted to mass density using a two-segment linear function according to Schneider et al. [18]. SIMIND simulations of both activity distributions were performed as described before, with the detector orbit adjusted to the actual non-circular orbits of the physical phantom measurements.

All SPECT/CT reconstruction in this work was performed based on 120 projections using OS-EM with 6 iterations and 8 subsets, employing compensation for attenuation and scatter using the ESSE method [19]. To convert the reconstructed counts into activity concentration, an image calibration factor (unit: counts-per-second-per-Megabecquerel) was determined as described in [20] based on the physical SPECT/CT measurement of the Jaszczak phantom described above. For these reconstructions, additional metrics were used to quantify the image quality. For the reconstructions of the Jaszczak phantom, the signal-to-noise ratio (SNR) was calculated in a cubic VOI (1519 mL) inside the cylinder:

2 $${\text{SNR}} = \frac{{\overline{A}}}{{\sigma_{{\text{A}}} }}$$

where $\overline{A}$ is the mean activity concentration in the VOI and $\sigma_{{\text{A}}}$ is the standard deviation of the voxel-to-voxel activity concentrations within the VOI. For the reconstructions of the NEMA phantom, the recovery, defined as the SPECT-derived activity in the spheres divided by the activity derived at phantom preparation, was calculated for all six spheres. The SPECT-based activity in each sphere was calculated by multiplying interpolated SPECT/CT reconstructions (nearest-neighbor interpolation, 256 × 256 matrix) with a binary mask (256 × 256 matrix), which was created using the known positions and dimensions of the spheres inside the phantom.

## Results

### Influence of noise

**Fig. 5:**
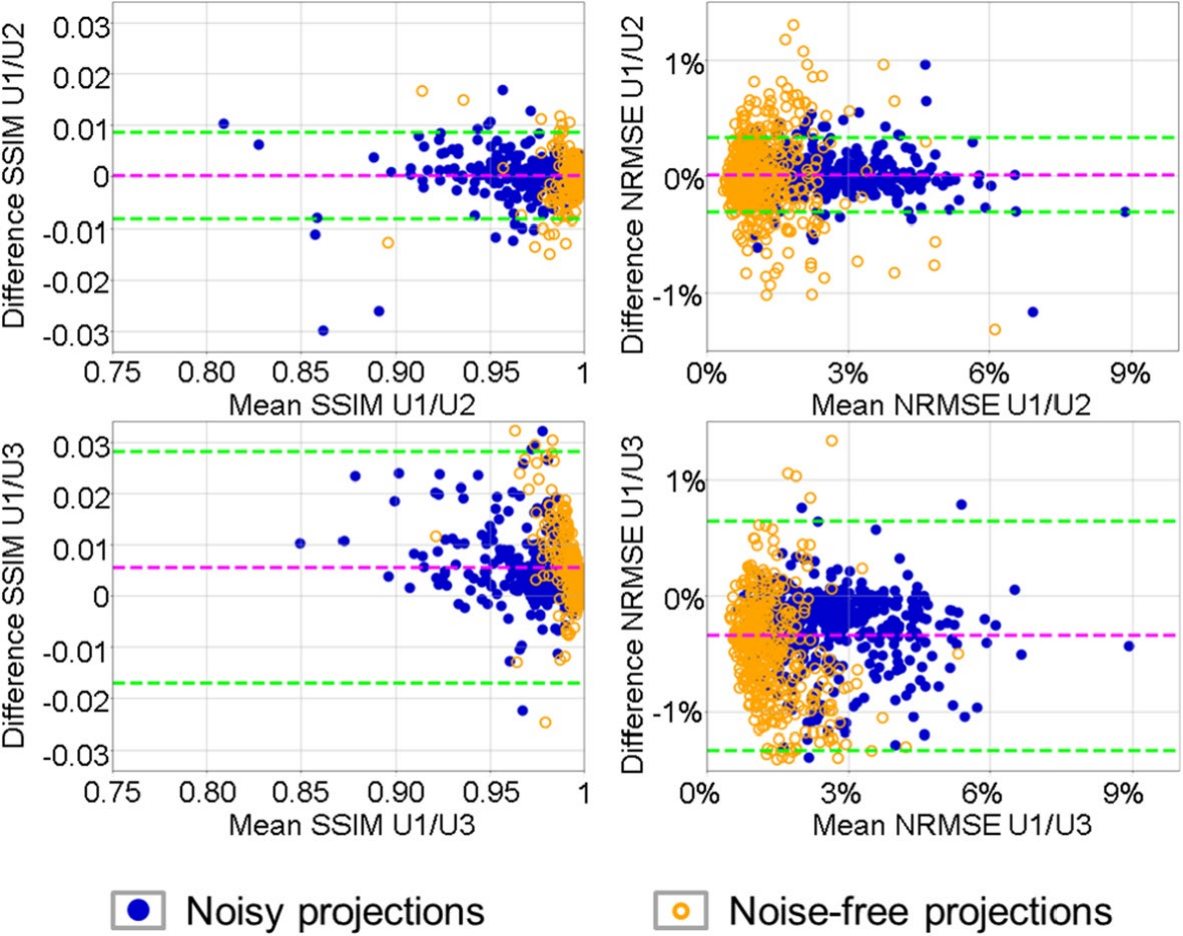
Bland–Altman plots illustrating the performance of u-nets U2 and U3 in comparison with U1. Bland–Altman plots for the differences in SSIM and NRMSE between synthetic projections of u-net U1 and U2 (top) and U3 (bottom). Blue circles depict the differences for the noisy projections, while the orange circles depict the differences for the noise-free projections. The magenta lines indicate the mean difference of the values for the noisy projections, and the green lines indicate the 95% confidence interval

Table 3 summarizes the quantitative performance analysis of the u-nets. For each activity distribution (total of 500) in the test dataset, the mean SSIM and the mean NRMSE were calculated over all synthetic projections (e.g., 60 projections for U1, 30 projections for U4, and 120 projections for U7). Then, the mean over these means was calculated for all 500 test datasets. For u-nets U1 and U2, the synthetic projections show no significant difference in SSIM (*p* = 0.18 and *p* = 0.69, paired two-sided Wilcoxon signed-rank test) and NRMSE (*p* = 0.15 and *p* = 0.68, paired two-sided t test) to noise-free and noisy projections; this is also highlighted by the Bland–Altman plots in Fig. 5 (both upper graphs). For both networks, however, the difference between synthetic and noise-free projections was significantly smaller than the difference between synthetic and noisy projections (*p* < 0.001). Moreover, the Bland–Altman plots show that there is no difference in the performance for both the u-nets trained with different noise realizations visible. When comparing the performance of the u-nets U1 and U3, it becomes apparent that using a larger training data set results in better SSIM and NRMSE values. Furthermore, in the Bland–Altman plots the noise-free projections (orange) demonstrate a smaller NRMSE and an SSIM closer to unity than the noisy projections (blue).

**Table 3:** Analysis of the u-net performance

Abbreviation	Synthetic versus noisy projections		Synthetic versus noise-free projections	
	SSIM	NRMSE	SSIM	NRMSE
U1	0.979 (0.025)	2.45% (1.18%)	0.995 (0.011)	1.19% (0.75%)
U2	0.979 (0.026)	2.44% (1.19%)	0.995 (0.011)	1.19% (0.79%)
U3	0.973 (0.033)	2.79% (1.29%)	0.989 (0.022)	1.85% (1.17%)
U4	0.978 (0.024)	2.52% (1.25%)	0.994 (0.007)	1.29% (0.75%)
U5	0.978 (0.024)	2.53% (1.20%)	0.994(0.008)	1.32% (0.68%)
U6	0.978 (0.025)	2.56% (1.53%)	0.994 (0.009)	1.37% (0.75%)
U7	0.982 (0.018)	2.24% (1.10%)	0.997 (0.006)	1.04% (0.61%)

Mean SSIM and NRMSE values between synthetic projections and noisy/noise-free projections, respectively. Data in parentheses are standard deviations

### Influence of the size of the training dataset on the u-net performance

Based on NRMSE and SSIM, u-net U3, which is based on a considerably smaller training dataset than the other u-nets, produced significantly worse synthetic projections than the networks trained with larger datasets (*p* < 0.001 when compared against U1 or U2). This observation is underlined numerically by the mean SSIM (smallest value for U3) and NRMSE (highest value for U3) values given in Table 3 as well as visually by the increased difference between the results of U1 and U3 in Fig. 5.

### Influence of the number of input projections and rotation angle

**Fig. 6:**
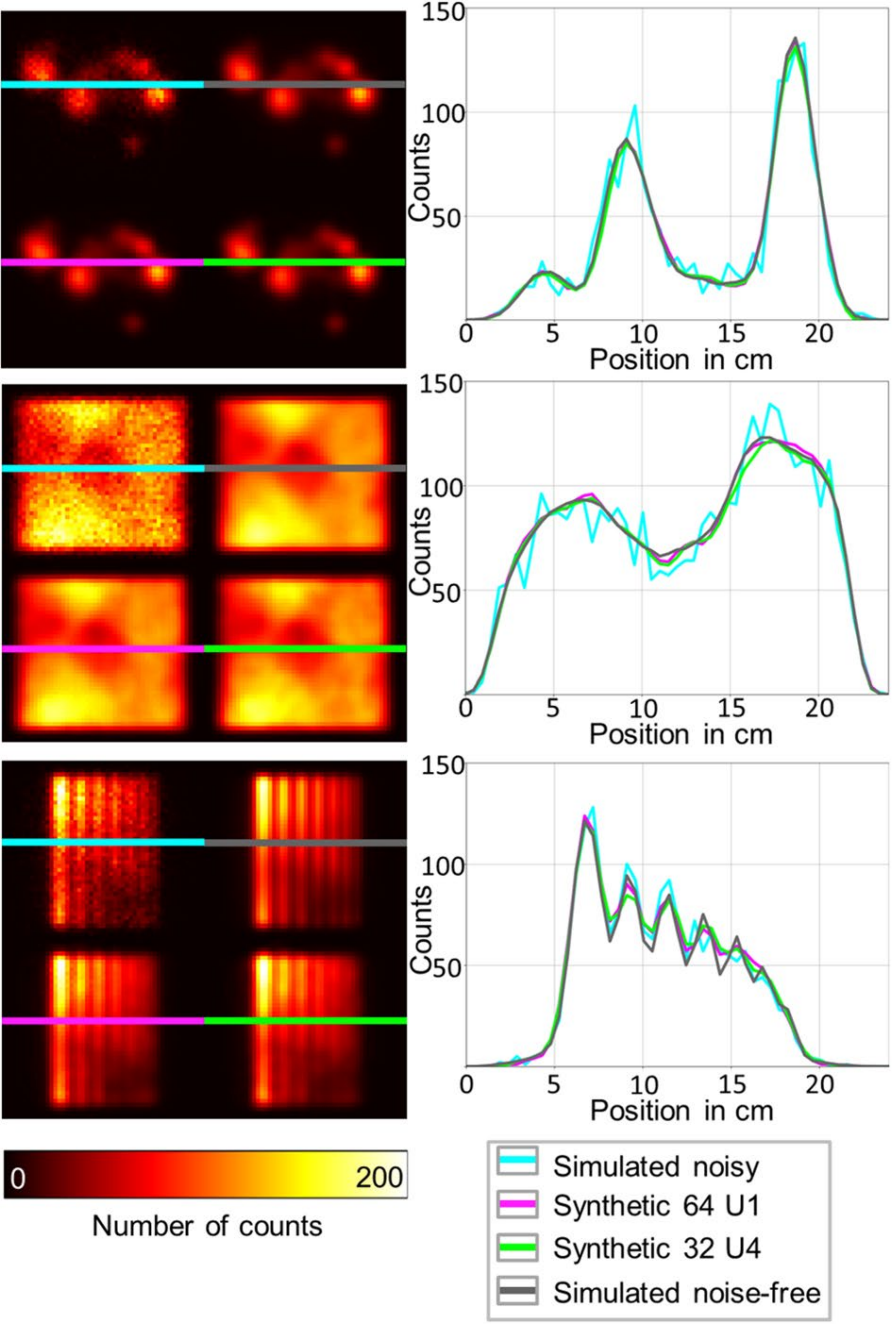
Comparison of simulated noisy and noise-free projections with synthetically generated projections. The synthetic projections were generated by u-nets U1 (magenta) and U4 (green), respectively. From top to bottom, the projections of the following activity distributions are shown: random shape, hot Jaszczak with cold random shapes, stripe pattern. The graphs on the right show cross sections through the projections along the colored lines

No significant differences in NRMSE and SSIM values were observed between u-nets U4, U5, and U6, which perform a 3°, 6°, and − 3° rotation, respectively, for 32 input projections (SSIM and NRMSE, *p* > 0.05 for u-nets U4, U5, and U6 noisy and noise-free projections). When comparing the synthetic projections generated based on networks U1/U2 (64 input/output projections) to the synthetic projections of U4/U5/U6 (32 input/output projections), the u-nets with a higher number of input projections perform significantly better than the u-nets with a lower number of input projections (*p* < 0.001). Figure 6 shows synthetic projections created by u-nets U1 and U4. The synthetic projections created by both u-nets (green and magenta) show a high visual similarity to the noise-free projections (gray).

### Analysis of the u-net for denoising the projections

The SSIM and NRMSE for U7, which was trained only to denoise the 120 input projections, are shown in Table 3. This u-net achieves significantly higher SSIM and lower NRMSE values than the other u-nets when compared to both the noisy and the noise-free projections.

### Influence of the detector orbit

Table 4 shows the mean SSIMs and NRMSEs between the synthetic projections and the noisy projections for the original and the four radially expanded detector orbits. While there are no significant differences in the u-net’s performance for small deviations of the detector orbit (2 cm and 4 cm radial expansion compared to the original detector orbit; 2 cm: SSIM, *p* = 0.47; NRMSE, *p* = 0.95; 4 cm: SSIM, *p* = 0.19; NRMSE, *p* = 0.10), the u-net performs significantly worse for larger radial expansions (SSIM/NRMSE, *p* < 0.01 for 8 cm and 16 cm). As expected, the largest expansion also has the lowest SSIM and the highest NRMSE values, respectively.

**Table 4:** Analysis of detector orbit deviations

	Original orbit	Orbit + 2 cm	Orbit + 4 cm	Orbit + 8 cm	Orbit + 16 cm
SSIM	0.979 (0.025)	0.980 (0.024)	0.979 (0.026)	0.978 (0.026)	0.976 (0.030)
NRMSE	2.45% (1.18%)	2.43% (1.21%)	2.46% (1.23%)	2.50% (1.22%)	2.63% (1.26%)

Mean SSIM and NRMSE values between synthetic projections and noisy projections for trajectories of increasing deviations. Data in parentheses are standard deviations

### Comparison between simulated and physical phantom measurements

**Fig. 7:**
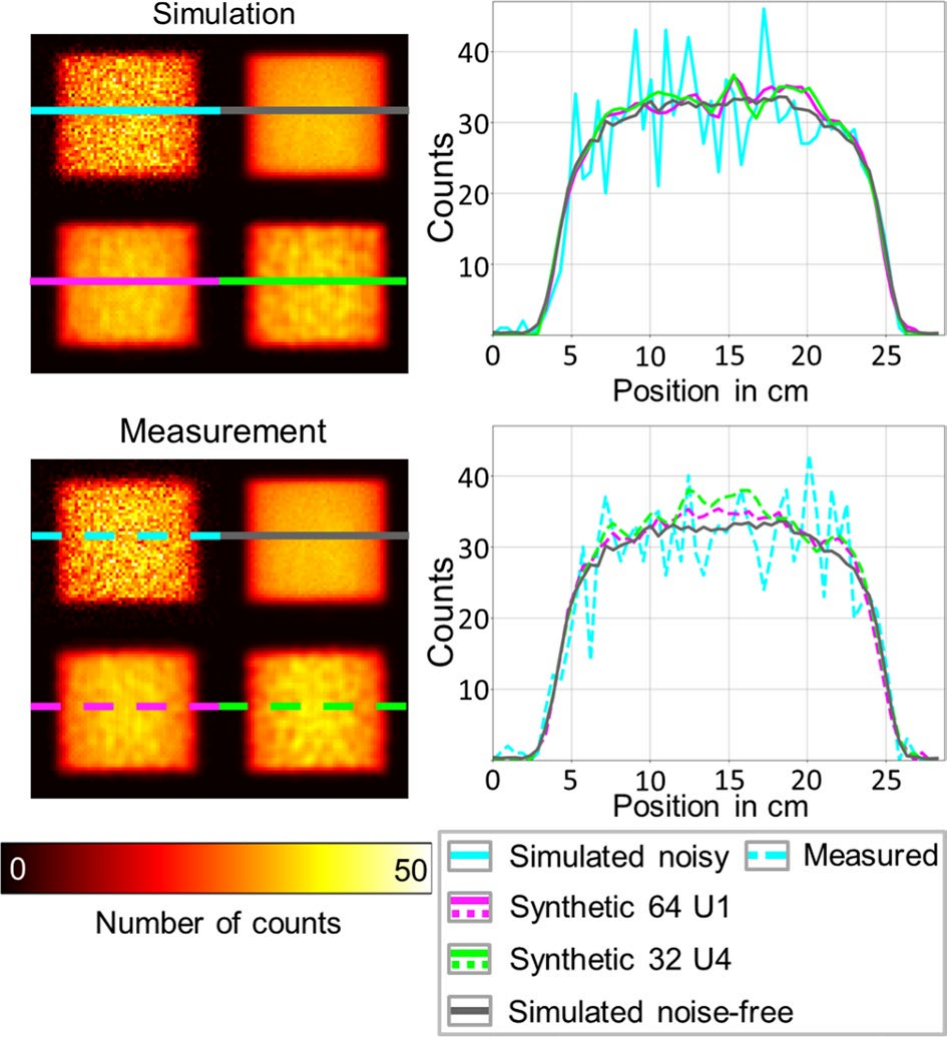
Comparison of measured and simulated projections of a filled Jaszczak cylinder with synthetic projections. The synthetic projections were generated by u-nets U1 (magenta) and U4 (green), respectively. The right graphs each show the cross section through the projections along the colored lines, where the solid curves represent the simulations and the dashed curves represent the measurements. The cross section through the noise-free (simulated) projection is also shown for both the measured and the simulated projections (solid gray curve)

Example projections of the simulated and the physical phantom measurements of the Jaszczak phantom, together with the corresponding synthetic projections, are shown in Fig. 7. The corresponding results for the NEMA Phantom can be found in Additional file 1. The related SSIM and NRMSE values between the projections are given in Tables 5 (differences to measured projections) and 6 (differences to simulated noisy projections). For both phantoms, there is a good agreement between measured and simulated projections (Jaszczak: SSIM, 0.965; NRMSE, 3.40%; NEMA SSIM, 0.990; NRMSE 0.93%). This shows that MC simulations can generate realistic SPECT projections. The synthetic projections of both u-nets are visually and numerically more similar to the noise-free simulated projections than to the measured or noisy simulated projections for both phantoms. This statement is supported by the higher SSIM and lower NRMSE values in Tables 5 and 6. Moreover, in Fig. 7, it can be seen that the synthetic projections are visually more similar to the noise-free projections than to the noisy projections.

**Table 5:** Analysis of physical phantom measurements

Projections		Synthetic 64 U1		Synthetic 32 U4		Measured	
		SSIM	NRMSE (%)	SSIM	NRMSE (%)	SSIM	NRMSE (%)
Jaszczak	Simulated noise-free	0.993	3.14	0.989	3.74	0.973	7.11
	Measured	0.979	5.85	0.978	5.71	1	0
NEMA	Simulated noise-free	0.992	0.49	0.992	0.50	0.986	0.72
	Measured	0.987	0.59	0.989	0.58	1	0

SSIM and NRMSE values between synthetic projections generated based on measured projections and simulated noise-free and measured projections, respectively

**Table 6:** Analysis of the simulations of the physical phantom measurements

Projections		Synthetic 64 U1		Synthetic 32 U4		Simulated noisy	
		SSIM	NRMSE (%)	SSIM	NRMSE (%)	SSIM	NRMSE (%)
Jaszczak	Simulated noise-free	0.998	1.94	0.998	2.20	0.973	7.70
	Simulated noisy	0.975	7.70	0.975	7.56	1	0
NEMA	Simulated noise-free	0.997	0.43	0.995	0.49	0.990	0.93
	Simulated noisy	0.987	0.94	0.985	1.05	1	0

SSIM and NRMSE values between synthetic projections generated based on simulated noisy projections and simulated noise-free and noisy projections, respectively

**Fig. 8:**
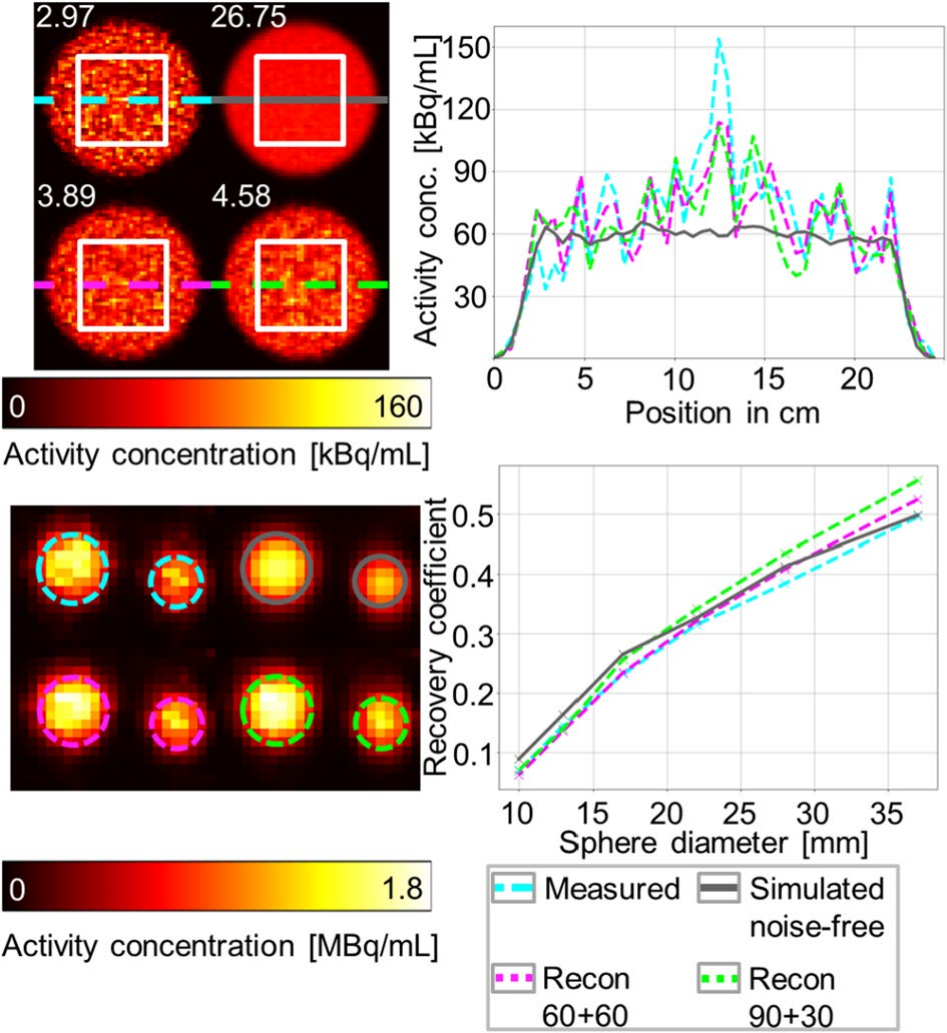
Analysis of physical phantom measurements. Left: Transversal slices of SPECT/CT reconstructions of the filled Jaszczak (top) and the NEMA phantom (bottom) using 120 projections. For the sake of clarity, only a section containing the two largest spheres are shown for the NEMA phantom. The missing projections for Recon 60 + 60 (magenta) and Recon 30 + 90 (green) were generated using u-nets U1 and U4–U6, respectively. The white numbers in the upper left panel are the signal-to-noise ratios calculated in the white boxes. For the Jaszczak phantom, the cross section of the activity distribution along the colored lines is shown on the right. For the NEMA phantom, the recovery of the spheres is shown on the right

Figure 8 shows SPECT/CT reconstructions of the phantom measurements. To imitate a SPECT acquisition accelerated by factors of 2 and 4, two u-net-based reconstructions were performed using only 60 (every other) and 30 (every fourth) measured projections, respectively. In order to compensate for the lost information, the missing projections were replaced by synthetic projections generated using u-nets U1 (60 synthetic projections) and U4–U6 (30 synthetic projections each, 90 in total), respectively. The reconstructions based on this mixed dataset of synthetic and measured projections are referred to as Recon 60 + 60 and Recon 30 + 90 for convenience. For the Jaszczak phantom reconstructions, the same observations as for the underlying projections can be made: The synthetic projections feature reduced noise, which is visually evident from higher signal-to-noise ratios (SNRs) for Recon 60 + 60 (about 30% increase) and Recon 30 + 90 (about 50% increase) when compared to the original SPECT. Interestingly, the SNR increases if more synthetic projections are included in the reconstruction (e.g., when comparing Recon 60 + 60 to Recon 30 + 90). The findings for the NEMA phantom are similar. For the reconstructions, the recovery of the two largest spheres is higher using synthetic projections than using the original projections only. For the smallest spheres, the reconstruction using the noise-free simulated projections yielded the best recovery. It should be noted that no resolution recovery was used for the reconstruction and thus lower recovery values are to be expected.

## Discussion

In this study, a large dataset of SPECT projections was produced by Monte Carlo simulations to analyze the performance of a methodology for AI-based generation of synthetic projections. By the use of rotated and randomly arranged random shapes in combination with a method for generating activity heterogeneity, a wide range of activity distributions in a clinically relevant range was generated and used as input to the MC simulations.

By using the simulated data, the problem of sparsity of clinical dataset for training of the u-net was circumvented. In this regard, the comparison of the performance of u-nets U1 (trained with 9,500 SPECT simulations) and U3 (trained with 400 SPECT simulations) showed a significant improvement if a larger training dataset was used. This indicates that results from previous studies [6, 21, 22] could be improved with a larger training set. As shown here, such an expanded dataset could, for example, be achieved based on MC simulated data rather than only relying on the typically small number of clinically available images. Although several previous studies applied large training datasets for deep learning-based improvement in SPECT imaging, these datasets were not realistic enough to be transferred to clinical SPECT systems. For example, Shao et al. [23, 24] and Chrysostomou et al. [25] each used a large dataset consisting of analytically derived digital phantoms that showed limited physical effects such as photon scatter, attenuation, and non-perfect collimation. (Shao et al. includes attenuation and non-perfect resolution in 2D.) In contrast, these physical effects are taken into account in our MC simulations, resulting in much more realistic projections. This statement is supported by the good agreement between simulations and measurements of the two physical phantoms. Another disadvantage of most training datasets published so far is that although some of them consisted of different shapes, the activity concentration of each of these shapes was uniform. In contrast, the dataset used in this study features a heterogeneous activity concentration distribution, making it much more realistic and comparable to clinical SPECT data.

Despite the significant improvement, there are still some limitations and simplifications in the simulated training dataset presented in this work that need to be adjusted before a potential clinical application: First, the localization of the activity in the input data is restricted to the region of the Jaszczak cylinder. Therefore, there could be deviations and artifacts for input activity distributions exceeding the cylinder dimensions. Another shortcoming is that photon attenuation in all simulations was based on the uniformly water-filled cylinder, which may differ from the clinical situation, e.g., imaging of the thorax region. Another aspect that might be looked at in more detail in the future is the influence of the detector orbit, which can strongly differ from the orbit of the NEMA phantom in clinical situations. In our small, NEMA orbit-based, sub-study, however, there was no significant difference in u-net performance for small orbit variations (radial expansion by 2 cm and 4 cm). For larger expansions (8 cm and 16 cm), however, we found an increasing impact on the u-net performance. Because the mean patient detector orbit is similar to the NEMA trajectory radially expanded by 4 cm (Fig. 5), this issue is not expected to be a major problem for the average patient. However, for patients of very large body sizes or in the event that auxiliary equipment is located near the patient, large deviations in detector orbit can potentially affect the performance of the u-net.

Since no significant differences in SSIM and NRMSE values were found between u-nets U1 and U2, we conclude that the noise realization of the training datasets only has a negligible effect on the u-net performance. The fact that all synthetic projections were more similar to the noise-free projections than to the noisy projections indicates that the u-net has a denoising effect in addition to the rotation. This effect can also be identified visually based on the good agreement between the magenta and green curves (synthetic projections generated by U1 and U4) and the gray curve (noise-free projection) in Fig. 6. This denoising effect of u-nets has also been described in the literature. For example, Ulyanov et al. showed that u-nets tend to amplify the signal and suppress noise [26]. In this study, we were able to show that this also applies to the Poisson noise of SPECT projections and that the projections denoised by the u-net are very similar to the noise-free projections obtained directly from the simulations.

Furthermore, the u-net performance decreased with a decreasing number of input projections. This might lead to the conclusion that it may be advantageous to acquire more projections with a lower measurement time per projection. However, the effect is most likely caused by the larger total signal when acquiring more projections for a constant projection time. Hence, it might be of interest to also study the performance of u-nets for a varying number of input projections, but for a constant total acquisition time, to get a more fair comparison with respect to total acquired signal. However, such aspects were not pursued in the current study. Another option for a future improvement in the methodology is the training of u-nets with an unbalanced number of input versus output projections (e.g., 30–90 or 15–105). Although initial attempts showed promising results, further analysis will be required, because the use of fewer input projections might increase the negative impact of trajectory differences.

The denoising effect is further illustrated by the best agreement between the u-net output and the noise-free initial projections, which was obtained for u-net U7, which is designed solely for denoising the projections (i.e., without any rotation). This leads to the conclusion that the rotation additionally performed by u-nets U1 to U6 introduces additional deviations between the ground-truth projections and the u-net output that cannot perfectly be corrected by the u-net. This rotation is not applied in case of u-net U7, however, resulting in the best performance of all networks.

Visually, the u-net performance becomes worse as the feature size approaches the resolution of the imaging system. This can be seen from the projection of the stripe pattern phantom in Fig. 6. A smoothing of the signal for the synthetic projections can be seen for the smallest stripes.

The analysis of the phantom measurements shows that, despite having been trained with SPECT simulations only, these u-nets can be applied to generate realistic synthetic projections for physical phantom SPECT/CT measurements. This, in combination with the good agreement between simulated and measured projections demonstrated in Fig. 7, indicates that the level of realism of the simulations using the SIMIND MC program was sufficient. Based on the good agreement between the synthetically generated and measured projections, it was shown that u-nets trained on simulated data can also be used for measured data. However, it should also be noted that the activity distribution in our dataset is limited to a Jaszczak cylinder. In cases where activity is present outside the Jaszczak dimensions, errors in the synthetic projections may occur. For application to clinical data, the diversity of the dataset may have to be further increased (e.g., by using differently shaped scatter media). Alternatively, the trained networks can also serve as a basis for training patient data using transfer learning. In general, while most published u-nets are made available with suggested parameter settings, a large number of hyperparameters can be tuned in the process of setting up and optimizing the performance of convolutional neural networks. Instead, the focus of this work lied primarily on various aspects of the SPECT imaging part of the methodology (e.g., noise, number of input data, and detector orbit). Tuning of hyperparameters or the deployment of entirely different network architectures might lead to an additional improvement in the network performance.

In summary, we show that realistic MC SPECT simulations can and should be applied to assess the performance of u-nets trained to generate SPECT projections. Moreover, simulated data could also be added to the typically small clinical datasets (e.g., using transfer learning) to improve the performance of such u-nets. It should be added, however, that a prerequisite for this is that the simulations are replicated with adequate accuracy in modeling the clinical system (e.g., energy resolution, collimator) and measurement conditions (e.g., the detector orbit).

## Conclusion

In this study, a large dataset of simulated SPECT projections of heterogeneous random shapes for evaluation of a deep learning-based generation method of SPECT projections was generated using the Monte Carlo simulation program SIMIND. We found that the size of the training dataset has a significant impact on the u-net performance for generation of intermediate projections in SPECT. In addition, a denoising effect by the u-net could be numerically shown in addition to the rotation. Here, the noise representation of the training datasets had no significant influence on the u-net performance. Regarding the detector orbit, small deviations did not show a significant deterioration in u-net performance. Most importantly, the u-nets trained solely based on MC simulated SPECT data could successfully be applied to physical phantom measurements, which could considerably increase the amount of available training data in future applications.

## Supplementary Information

**Additional file 1. Figure S1.** Schematic illustration of the u-shaped convolutional neural network employed in this study (example 64 → 64 projections). **Figure S2.** Comparison of measured and simulated projections of a NEMA phantom with synthetic generated projections by u-nets U1 and U4. The right graphs each show the cross section through the projections along the colored lines, where the solid curves represent the simulations and the dashed curves represent the measurements. The noise-free projection (simulation) is also shown for both the measured and simulated projections (gray curve). **Figure S3.** Training and validation loss curves for the training of u-nets U1 and U3. Since a different number of training epochs and data were selected for the training of both networks, the relative training step is given on the x-axis. Therefore, a training step of 100% corresponds to 60 epochs for u-net U1, while it corresponds to 200 epochs for u-net U2.

## Data Availability

The datasets used and/or analyzed during the current study are available from the corresponding author on reasonable request.

## Abbreviations

u-net: U-shaped convolutional neural network
RNT: Radionuclide therapy
SNR: Signal-to-noise ratio
AI: Artificial intelligence
MC: Monte Carlo
3D: Three-dimensional
MELP: Medium-energy low penetration
ReLU: Rectified linear unit
SSIM: Structural similarity index measurement
NRMSE: Normalized root-mean-squared error
