# Supplementary material for: Analysis of a deep learning-based method for generation of SPECT projections based on a large Monte Carlo simulated dataset
Source: EJNMMI Phys. 2022 Jul 19;9:47. doi: 10.1186/s40658-022-00476-w (PMC9296746; doi:10.1186/s40658-022-00476-w)
Supplement: Supplementary file 1 — Additional file 1. Figure S1. Schematic illustration of the u-shaped convolutional neural network employed in this study (example 64 → 64 projections). Figure S2. Comparison of measured and simulated projections of a NEMA phantom with synthetic generated projections by u-nets U1 and U4. The right graphs each show the cross section through the projections along the colored lines, where the solid curves represent the simulations and the dashed curves represent the measurements. The noise-free projection (simulation) is also shown for both the measured and simulated projections (gray curve). Figure S3. Training and validation loss curves for the training of u-nets U1 and U3. Since a different number of training epochs and data were selected for the training of both networks, the relative training step is given on the x-axis. Therefore, a training step of 100% corresponds to 60 epochs for u-net U1, while it corresponds to 200 epochs for u-net U2. [file 40658_2022_476_MOESM1_ESM.pdf]

SUPPLEMENTAL DATA

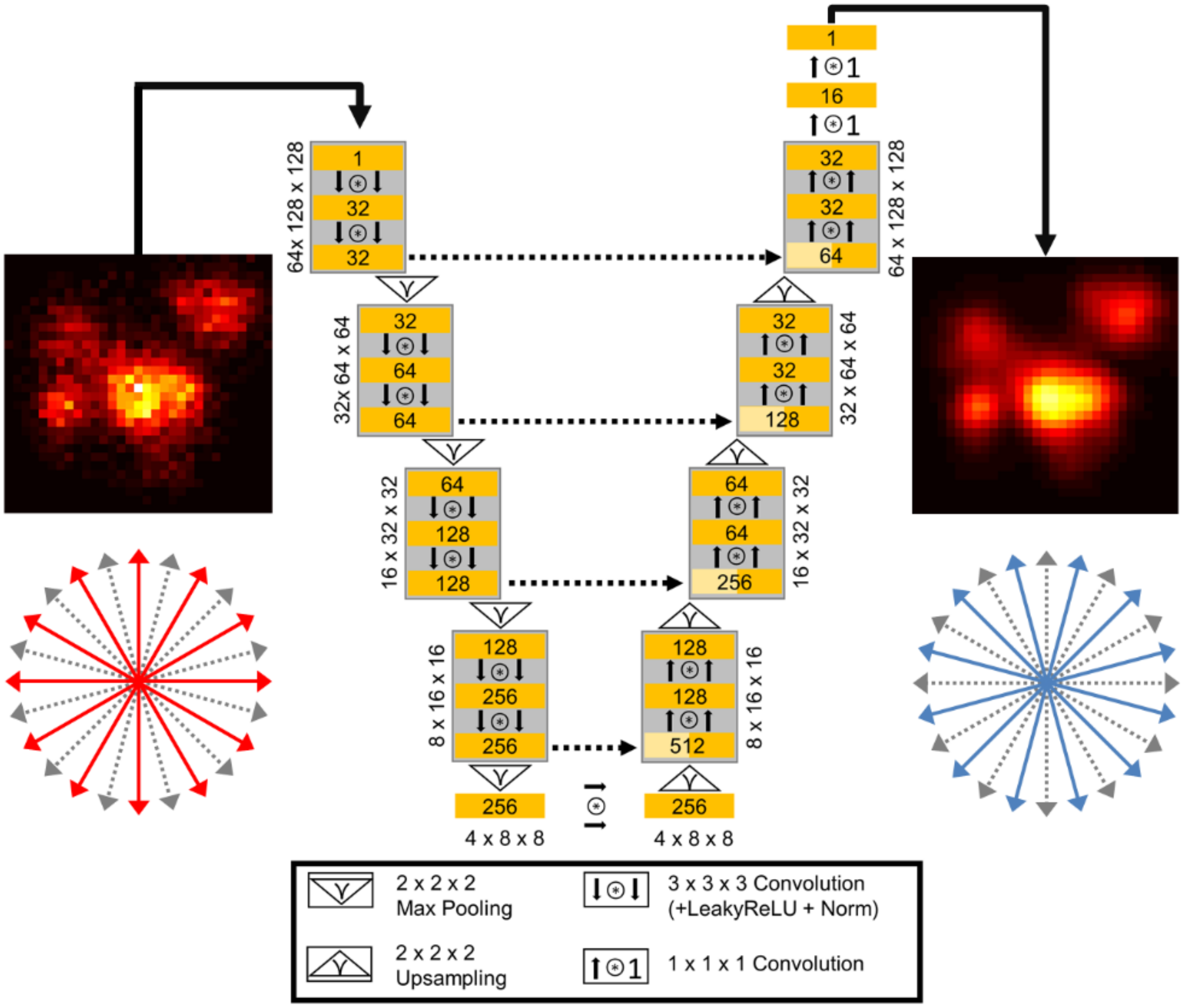

Figure 1. Schematic illustration of the u-shaped convolutional neural network employed in this study (example 64 → 64 projections).

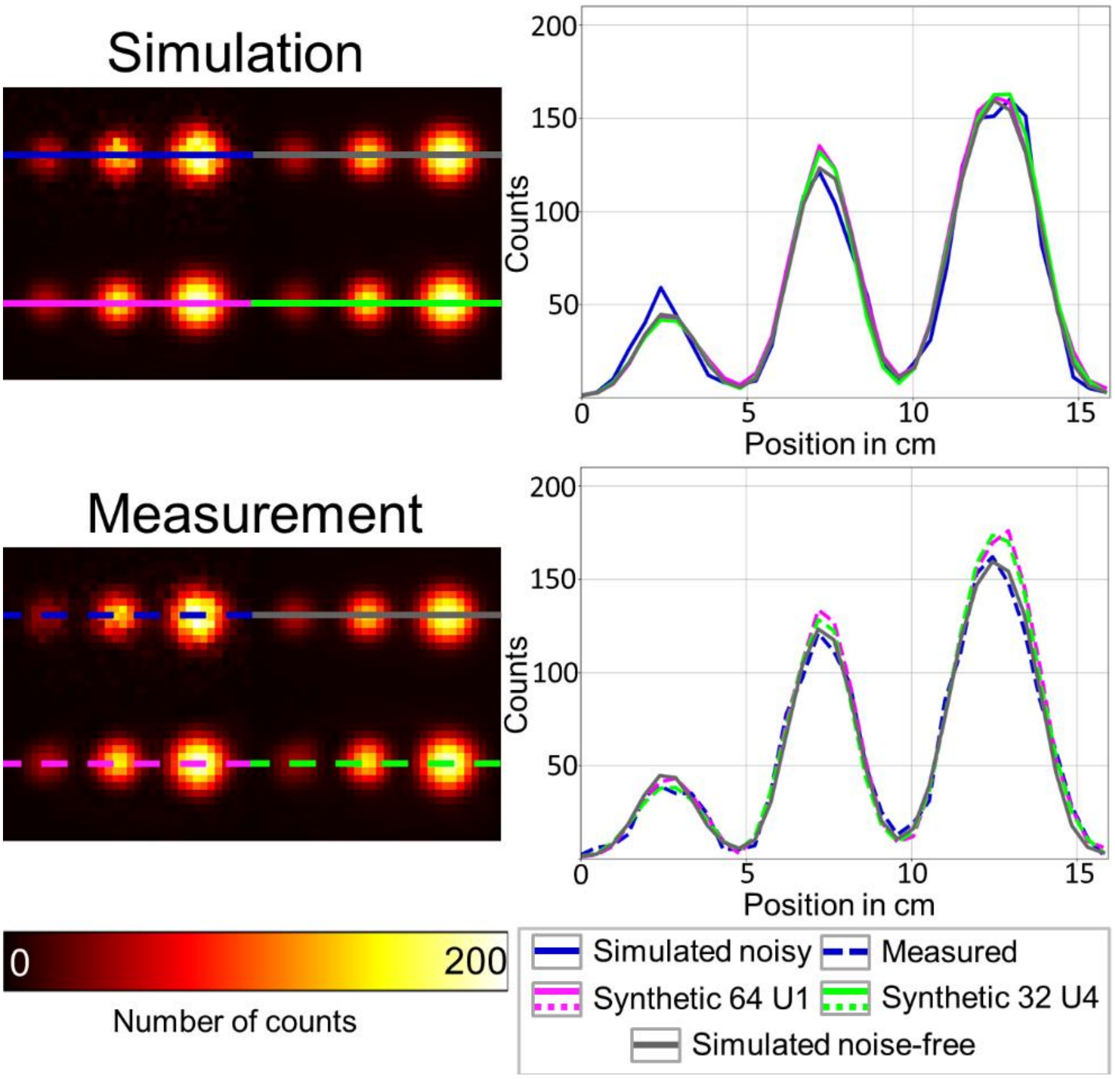

Figure 2: Comparison of measured and simulated projections of a NEMA phantom with synthetic generated projections by u-nets U1 and U4. The right graphs each show the cross-section through the projections along the colored lines, where the solid curves represent the simulations and the dashed curves represent the measurements. The noise-free projection (simulation) is also shown for both the measured and simulated projections (gray curve).

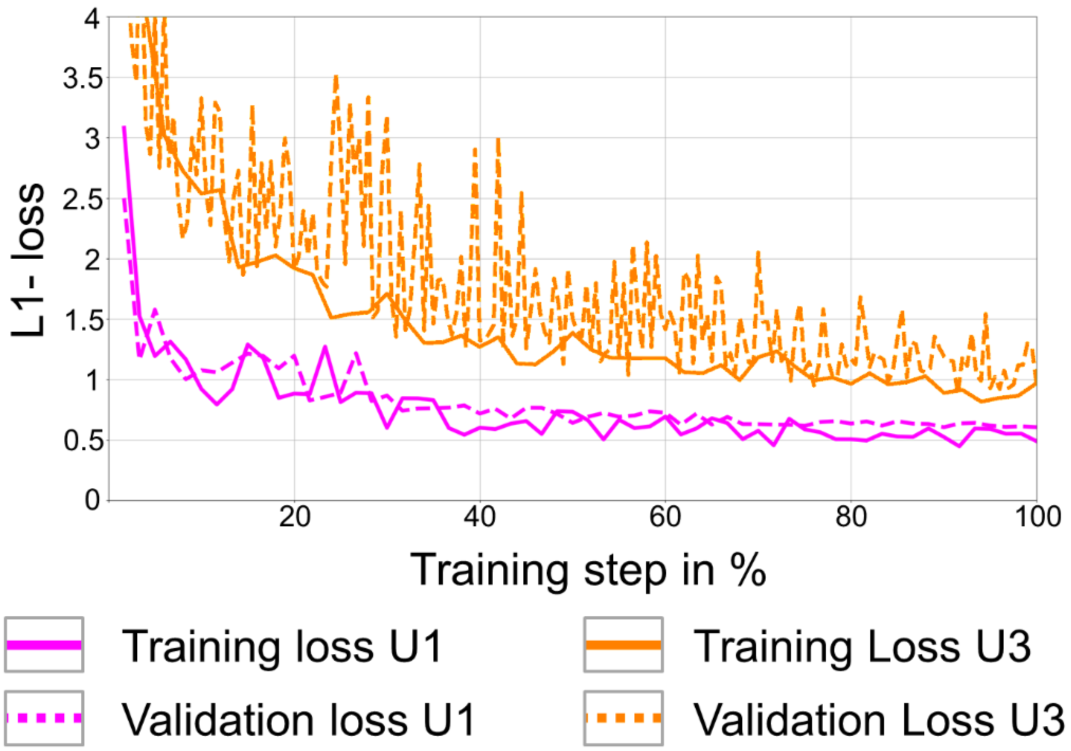

Figure 3: Training and validation loss curves for the training of u-nets U1 and U3. Since a different number of training epochs and data were selected for the training of both networks, the relative training step is given on the x-axis. Therefore, a training step of 100% corresponds to 60 epochs for u-net U1, while it corresponds to 200 epochs for u-net U2.
